# Variants of Oxidized Regenerated Cellulose and Their Distinct Effects on Neuronal Tissue

**DOI:** 10.3390/ijms222111467

**Published:** 2021-10-25

**Authors:** Joshua Kleine, Sandra Leisz, Chalid Ghadban, Tim Hohmann, Julian Prell, Christian Scheller, Christian Strauss, Sebastian Simmermacher, Faramarz Dehghani

**Affiliations:** 1 Medical Faculty, Institute of Anatomy and Cell Biology, Martin Luther University Halle-Wittenberg, 06112 Halle (Saale), Germany; Joshua.kleine@student.uni-halle.de (J.K.); Chalid.ghadban@medizin.uni-halle.de (C.G.); Tim.hohmann@medizin.uni-halle.de (T.H.); Faramarz.dehghani@medizin.uni-halle.de (F.D.); 2 Department of Neurosurgery, Medical Faculty, Martin Luther University Halle-Wittenberg, 06120 Halle (Saale), Germany; Julian.prell@uk-halle.de (J.P.); Christian.scheller@uk-halle.de (C.S.); Christian.strauss@uk-halle.de (C.S.); Sebastian.simmermacher@uk-halle.de (S.S.)

**Keywords:** cellulose applications, oxidized regenerated cellulose, Tabotamp^®^, Equicel^®^, Equitamp^®^, cell death, organotypic hippocampal slice cultures

## Abstract

Based on oxidized regenerated cellulose (ORC), several hemostyptic materials, such as Tabotamp^®^, Equicel^®^ and Equitamp^®^, have been developed to approach challenging hemostasis in neurosurgery. The present study compares ORC that differ in terms of compositions and properties, regarding their structure, solubility, pH values and effects on neuronal tissue. Cytotoxicity was detected via DNA-binding fluorescence dye in Schwann cells, astrocytes, and neuronal cells. Additionally, organotypic hippocampal slice cultures (OHSC) were analyzed, using propidium iodide, hematoxylin-eosin, and isolectin B_4_ staining to investigate the cellular damage, cytoarchitecture, and microglia activation. Whereas Equicel^®^ led to a neutral pH, Tabotamp^®^ (pH 2.8) and Equitamp^®^ (pH 4.8) caused a significant reduction of pH (*p* < 0.001). Equicel^®^ and Tabotamp^®^ increased cytotoxicity significantly in several cell lines (*p* < 0.01). On OHSC, Tabotamp^®^ and Equicel^®^ led to a stronger and deeper damage to the neuronal tissue than Equitamp^®^ or gauze (*p* < 0.01). Equicel^®^ increased strongly the number of microglia cells after 24 h (*p* < 0.001). Microglia cells were not detectable after Tabotamp^®^ treatment, presumably due to an artifact caused by strong pH reduction. In summary, our data imply the use of Equicel^®^, Tabotamp^®^ or Equitamp^®^ for specific applications in distinct clinical settings depending on their localization or tissue properties.

## 1. Introduction

Intraoperative bleeding, especially during neurosurgical interventions, can lead to massive secondary complications. The rising number of neurosurgical patients under anti-coagulative therapy increases the risk of perioperative hemorrhage [1]. Consequently, the application of hemostatic agents is an important option to handle intraoperative bleeding. Oxidized regenerated cellulose (ORC) is a commonly used absorbable hemostatic product that prevents bleeding and controls epidural oozing [2]. In a comprehensive review of topical hemostatic agents, ORC showed only moderate hemostatic effects, but good handling properties [3]. It did not stick to instruments, could easily be fitted to the tissue [2] and was completely absorbable within weeks [4]. Chemically, ORC consist of homo-polysaccharides and dinitrogen tetroxide. The oxidation of their hydroxyl groups to carboxyl acid groups forms a polyuronic acid [5]. Depending on the oxidized molecular position, different physico-chemical properties result and the efficiency of oxidation is influenced by factors like temperature and pressure [3]. The carboxylic acid groups are responsible for decrease in the pH value with additional bactericidal properties [6,7]. Hemostasis is achieved via different mechanisms. Absorption of blood results in slightly swelling and formation of a plug at the injury site. Furthermore, the acidity causes erythrocyte cell lysis, and the released hemoglobin reacts with acid to form hematin [3,8,9]. Over the last few decades, several absorbable hemostyptic materials have been developed based on natural and artificial sources. Cellulose is a natural source of ORC. In comparison, viscose is a semi-artificial regenerated cellulose, which is chemically identical to the native fiber, but has a different ordered surface in the elementary lattice (hydrate cellulose). Regardless their source, all products are subsumed as ORC. Several approved ORC are offered on the European market including Tabotamp^®^ (Ethicon, Johnson & Johnson, respectively Surgicel^®^ as its brand name in USA), Equicel^®^ and Equitamp^®^ (Equimedical). Tabotamp^®^ is established since 1959, whereas Equicel^®^ and Equitamp^®^ have been approved according to the EN ISO 10993 as a medical device since 2012. Recently, we demonstrated for Tabotamp^®^ a strong and local cytotoxic effect in several monolayer cell culture systems. pH-sensitive fluorescence microscope analyses revealed a strong pH gradient between the local pH drop and the surrounding media [10]. 

Based on these data, the present study was designed to compare the effects of Tabotamp^®^, Equitamp^®^ and Equicel^®^ on pH values, properties in aqueous solution, cell detachment, glial cell response, cell death and the extension of cellular damage in monolayer cell systems and in the dentate gyrus of the organotypic hippocampal slice cultures.

## 2. Results

### 2.1. ORC Products Showed Different Structures and Properties in Aqueous Solution

**Figure 1:**
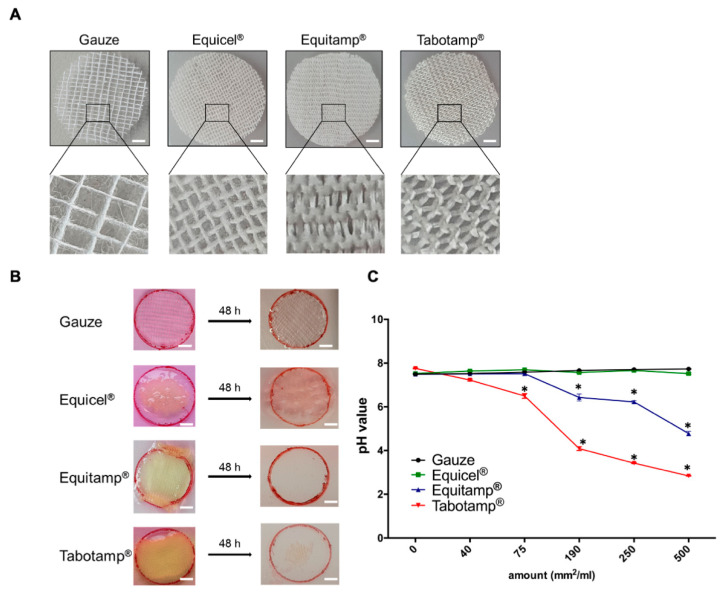
Comparison of ORC properties. Treatment materials with 22 mm diameter. In all experiments gauze served as non-oxidized, non-absorbable control. Gauze and Equicel^®^ contain frayed organized fibers, which are arranged in a square network structure. Equicel^®^ is an ORC based on natural cotton and contains collagen and fibrin. Equitamp^®^ and Tabotamp^®^ have a woven mesh structure. The viscose-based mesh of Equitamp^®^ has a higher density compared to Tabotamp^®^. Bar = 3 mm (**A**). Samples were wetted with DMEM to compare the dissolution and absorbability. Bar = 5 mm (**B**). The pH values of increasing amounts of gauze or ORC per milliliter supplemented DMEM. The graph shows the mean and SEM of three independent analyses. The significance was tested to the same amount of gauze (* *p* < 0.001) (**C**).

ORC showed strong structural differences. While gauze and Equicel^®^ consist of an ordered network based on frayed natural cotton fibers, Equitamp^®^ and Tabotamp^®^ were structured like a loose woven knit of smooth fibers. Equitamp^®^ had a higher density than Tabotamp^®^ (Figure 1A). 

To compare the absorbability, gauze and ORC were incubated for 48 h in aqueous solution (Figure 1B). The non-absorbable gauze showed no dissolution, whereas Equitamp^®^ and Tabotamp^®^ were nearly completely dissolved. Equicel^®^ formed a gelatin-like plug, which did not dissolve after 48 h. 

The different oxidation of ORC caused changes in pH value in aqueous solution (Figure 1C). Non-oxidized gauze led to an increase in pH value from 7.50 ± 0.02 without gauze to 7.73 ± 0.01 with 500 mm^2^/mL gauze. Even with large amounts of Equicel^®^, the pH value remained stable (pH 7.53 ± 0.01 without Equicel^®^ to 7.52 ± 0.04 with 500 mm^2^/mL Equicel^®^; *p* = 0.88). With smaller amounts of Equitamp^®^, the pH was stable (7.51 ± 0.04 with 40 mm^2^/mL and 7.52 ± 0.13 with 75 mm^2^/mL; *p* = 1.00). However, Equitamp^®^ reduced pH from 7.49 ± 0.02 to 6.43 ± 0.23, 6.22 ± 0.05 and 4.78 ± 0.10 with amounts of 190, 250 and 500 mm^2^/mL medium (*p* < 0.001). The strongest pH reduction was caused by Tabotamp^®^. Already amounts of 40 and 75 mm^2^ Tabotamp^®^ decreased the pH from 7.77 ± 0.02 to 7.28 ± 0.04 (*p* < 0.001) and 6.62 ± 0.10 (*p* < 0.001), respectively. Larger amounts of 190, 250, and 500 mm^2^/mL Tabotamp^®^ led to a strong acidic pH of 3.92 ± 0.09, 3.40 ± 0.09 and 2.84 ± 0.01 (*p* < 0.001) (Figure 1C).

#### Cell Detachment Analysis

**Figure 2:**
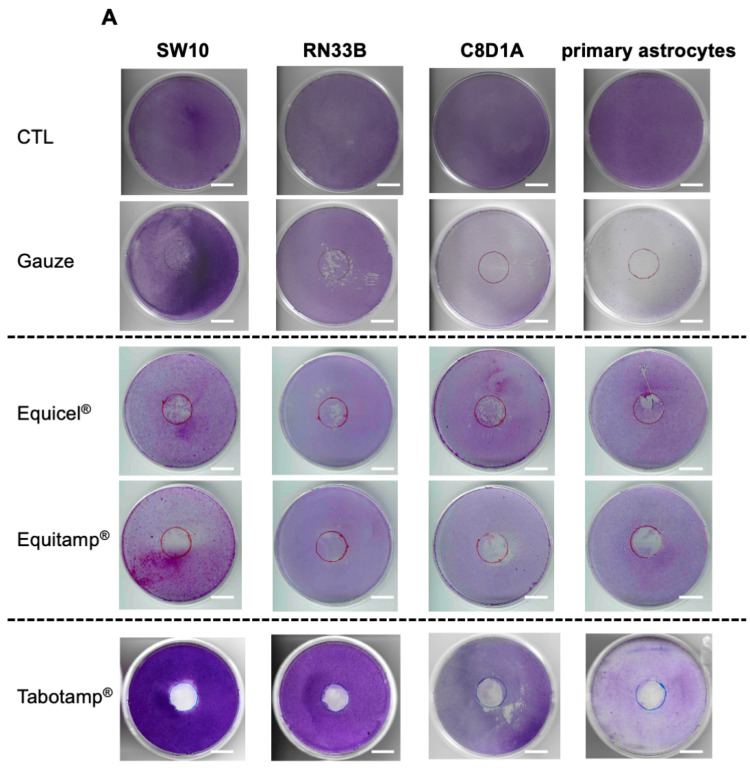

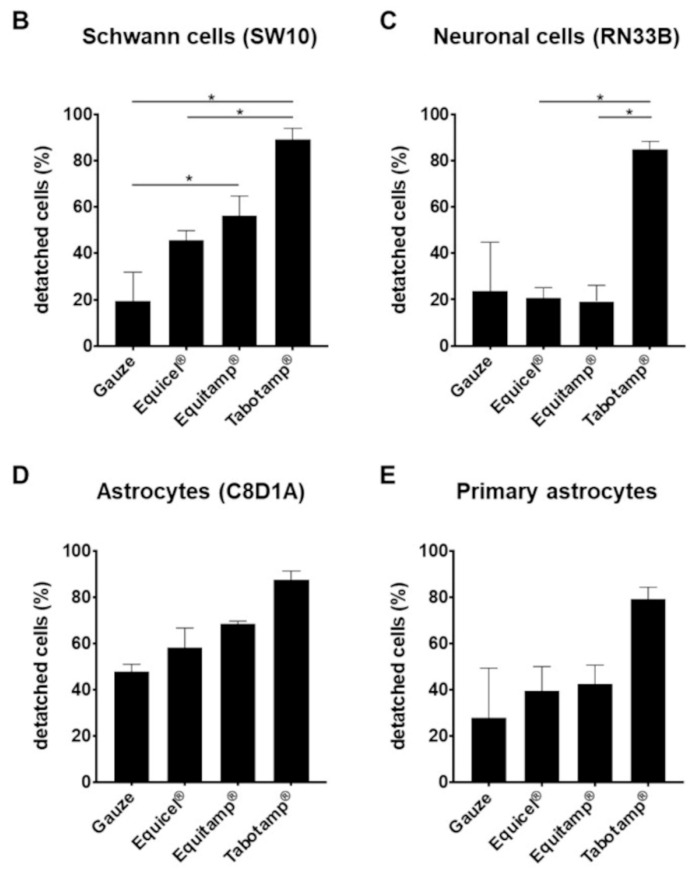
Detachment of cell monolayer after incubation with Equicel^®^ and Equitamp^®^. Exemplary crystal violet staining of each ORC and cell line or primary cells. The results of untreated cells (control, CTL) and after incubation with gauze and Tabotamp^®^ have already been published and are shown for comparison [10]. Cells were grown to complete confluence in 60 cm^2^ cell culture dishes and incubated with Equicel^®^ or Equitamp^®^. Bar = 2 cm (**A**). In addition, the means and SEM of quantified cell detachment analysis of three independent stained dishes from Schwann cells (**B**), neuronal cells (**C**), immortalized astrocytes (**D**) and primary astrocytic cultures (**E**) are shown (* *p* < 0.05).

Previously published results of cell detachment after incubation with Tabotamp^®^, gauze and untreated controls [10] were extended by experiments after incubation with Equicel^®^ and Equitamp^®^. Crystal violet staining was applied after incubation with the different ORC for 24 h (Figure 2A). The quantification of cell detachment after incubation of ORC on Schwann cells revealed that Equitamp^®^ (56.19% ± 8.55%) and Tabotamp^®^ (89.25% ± 4.74%) caused an increased damage of the cell monolayers compared to gauze (19.59% ± 12.30%, *p* < 0.05) (Figure 2B). In case of immortalized neuronal cells, astrocytes and primary astrocytes, Tabotamp^®^, Equicel^®^, and Equitamp^®^ showed no significant difference of cell detachment when compared to the gauze (Figure 2C–E; *p* > 0.05). 

### 2.2. Effect of Non-Oxidized Cellulose and ORC on Cell Viability

ORC-mediated cytotoxicity was measured via a fluorescence marked DNA-binding dye in Schwann cells, neuronal cells and astrocytes. Previously, we published the results for Tabotamp^®^ and included the data in the figures for a better comparison between the ORC [10]. 

**Figure 3:**
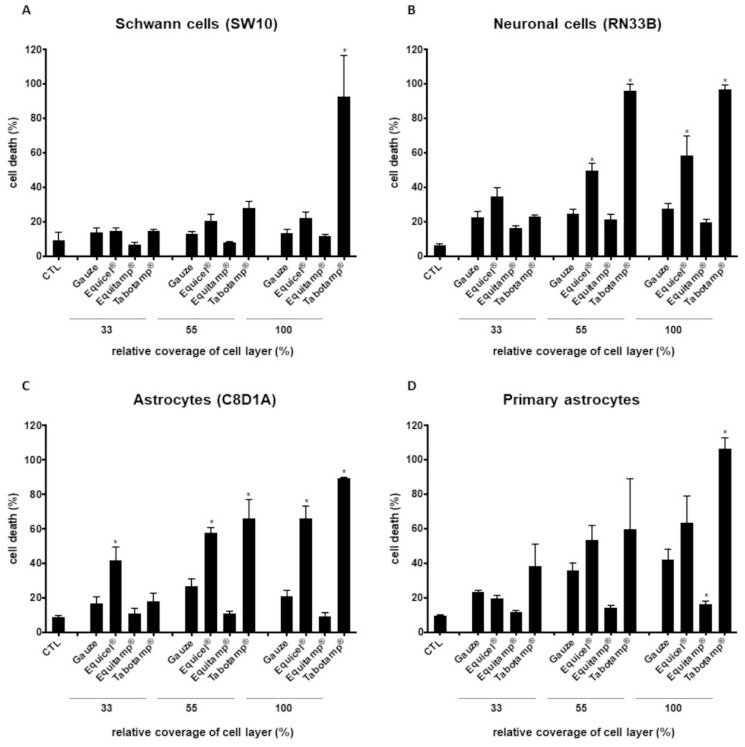
Detachment of cell monolayer after incubation with Equicel^®^ and Equitamp^®^. Equicel^®^ induced cell death of Schwann cells, neuronal cells and astrocytic cells. Quantification of cell death after 24 h using the CellTox Green assay. Schwann cells (SW10) (**A**), neuronal cells (RN33B) (**B**) and astrocytes (C8D1A and primary astrocytes) (**C**,**D**). ø 4 mm of non–oxidized cellulose (gauze), Equicel^®^ or Equitamp^®^ pieces correspond to 33% cell coverage, 5 mm pieces correspond to 55% cell coverage and 6 mm pieces correspond to 100% cell coverage. Signal of totally lysed cells of each cell type was set to 100% cell death. Cell culture medium without cells served as background control. Values are presented as means and SEM of four independent experiments. Statistical significance was analyzed with one-way ANOVA followed by Bonferroni’s test. Significance was accepted with *p* < 0.05: * significant to same amount gauze treated cells. For multiple statistical comparison of all groups, see Table S1. For better comparability the published Tabotamp^®^ cell death rates were included into the figures [10].

Compared to gauze, Equitamp^®^ and Equicel^®^ did not significantly affect cell death rates of Schwann cells. The highest cell death rates of Schwann cells were shown after incubation with Tabotamp^®^ (100% coverage, Figure 3A). Comparison of Equitamp^®^ with Equicel^®^ (100% coverage) caused a significantly higher cytotoxicity of Equicel^®^ (*p* = 0.018, Table S1).

Coverage with Equicel^®^ and Tabotamp^®^ showed an impaired neuronal cell survival, compared to gauze (55% and 100% coverage, *p* < 0.05, Figure 3B). Equitamp^®^ induced cell death rates were equivalent to gauze and significantly reduced compared to higher values of Equicel^®^ in neuronal cells (*p* < 0.05, Table S1). 

**Figure 4:**
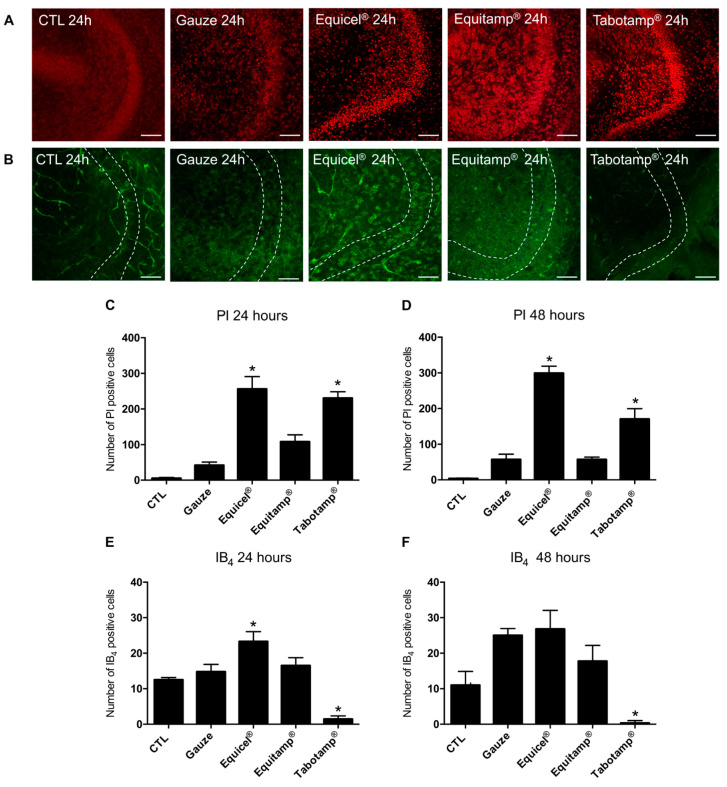
Effects of treatment with Equicel^®^, Equitamp^®^ and Tabotamp^®^ on cell viability and microglia activation in OHSC after 24 and 48 h. PI staining (red) of OHSC as recorded by confocal laser scanning microscopy representing dentate gyrus (DG) with granule cells (24 h results displayed) (**A**). Treatment with Equicel^®^ and Tabotamp^®^ led to intense red stained PI positive nuclei in DG regions. Equitamp^®^ caused a mild accumulation of PI in the cells of the DG. IB_4_ staining (green) of OHSC recorded by confocal laser scanning microscopy representing microglia cells and remaining vessels. In CTL, only isolated ramified microglia cells were visible. Treatment with Equicel^®^ led to increase of amoeboid microglia cells in the DG. Dotted lines mark the borders of GCL (**B**). Quantitative analysis of the number of PI positive degenerating cells in GCL (**C**,**D**). Equicel^®^ (* *p* < 0.001) and Tabotamp^®^ (* *p* < 0.01) induced a significant increase of PI positive cells in GCL 24 h after treatment compared gauze (**C**). Similar results were obtained for Equicel^®^ (* *p* < 0.001) and Tabotamp^®^ (* *p* < 0.01) compared to the OHSC treated with gauze after 48 h (**D**). Quantitative analysis of the number of IB_4_ positive microglia cells in GCL (**E**,**F**). After 24 h incubation with Equicel^®^, a significant increase in IB_4_ positive microglia in the DG compared to the gauze was observed (*, *p* < 0.05). A clearly reduced number of IB_4_ positive microglia was detected for Tabotamp^®^ compared to gauze (* *p* < 0.001) (**E**). After 48 h no significant difference in IB_4_ positive microglia cells between Equicel^®^ and gauze was detectable (**F**). A general increase in the number of microglia was found in treated groups compared to the results after 24 h (CTL compared to gauze: * *p* < 0.001 after 48 h). For Tabotamp^®^, a significant reduction of microglia was present after 48 h compared to gauze (* *p* < 0.001). For a statistical comparison of all groups, see Table S2. Bar = 100 µm.

Astrocytic cells demonstrated a high sensitivity to the treatment with ORC and even gauze (Figure 3C,D). Comparison between the ORC and gauze showed the highest cell death rates of immortalized astrocytes after incubation with Equicel^®^ (33%, 55% and 100% coverage, *p* < 0.002) and Tabotamp^®^ (55%, 100% coverage, *p* < 0.05, Figure 4C). In addition, for primary astrocytes Equicel^®^ increased cell death (55% coverage, *p* = 0.002, 100% coverage, *p* < 0.001) in comparison to gauze (Figure 3D), whereas Equitamp^®^ reduced cell death of primary astrocytes in comparison to gauze (55% and 100% coverage, *p* < 0.001).

Except for 33% coverage on immortalized astrocytes (C8D1A) the depicted Tabotamp^®^ data showed cell death rates that were comparable to or even exceeded those caused by Equicel^®^ (Figure 3C,D). 

Incubation with gauze caused a significant increase in cytotoxicity of astrocytes (C8D1A) in comparison to untreated cells (control, CTL) (55% coverage, *p* = 0.042). However, the cell death rates of Equitamp^®^ treated cells were comparable to CTL (Figure 3C, Table S1).

In addition, for primary astrocytes incubation with gauze increased cytotoxicity (55% and 100% coverage, *p* < 0.05, Figure 3D, Table S1).

### 2.3. Equicel^®^ and Tabotamp^®^ Generated Cellular Damage in the Dentate Gyrus of Organotypic Hippocampal Slice Cultures after 24 h

Untreated organotypic hippocampal slice cultures (OHSC) as well as slices treated with gauze displayed an excellent preservation of their cytoarchitecture. In CLSM only a few pycnotic propidium iodide (PI) positive nuclei were detected in granular cell layer (GCL) of the dentate gyrus (DG) (CTL: 5.4 PI positive cells/GCL, gauze: 42.8 PI positive cells/GCL) (Figure 4A,C). Treatment with Equicel^®^ and Tabotamp^®^ significantly increased the number of PI positive cells compared to gauze (Equicel^®^: 256.2 PI positive cells/GCL, *p* < 0.001; Tabotamp^®^: 230.4 PI positive cells/GCL, *p* < 0.01) (Figure 4A,C). No significant increase in the number of PI positive cells was found after treatment with Equitamp^®^ compared to gauze (Equitamp^®^: 108.3 PI positive cells/GCL, *p* > 0.05) (Figure 4A,C). 

To validate the extent of the overall damage indicated by the PI staining, the sections were assessed after staining with hematoxylin and eosin (HE). Treatment with Equicel^®^ and Tabotamp^®^ showed a clearly destroyed cytoarchitecture with pycnotic cell nuclei in the DG (Figure S1). The damage not only included superficial cell layers but also reached deep. The neuronal injury was consistently present in the z-stacks across all OHSC sectional images for both Equicel^®^ and Tabotamp^®^. With a distance between the optical sections of 5 µm and about 10–12 cuts per OHSC, a local, trans-sectional damage of at least 60 µm depth into the neuronal tissue was found.

### 2.4. The Increased Cellular Damage of Equicel^®^ and Tabotamp^®^ Persisted after 48 h in OHSC

To investigate the extent of the damage over time, the number of PI positive cells was analyzed after 48 h. In case of the untreated control and after incubation with gauze, a well-preserved cytoarchitecture and a low number of PI positive cells (CTL: 3.4 PI positive cells/GCL, gauze: 57.6 PI positive cells/GCL) were detected (Figure 4D). Equicel^®^ and Tabotamp^®^ caused a rising number of PI positive cells compared to gauze (Equicel^®^: 299.4 PI positive cells/GCL, *p* < 0.001; Tabotamp^®^: 170.4 PI positive cells/GCL, *p* < 0.01) (Figure 4D). Equitamp^®^ showed no significant change in PI positive cells after 48 h compared to gauze (Equitamp^®^: 57.3 PI positive cells/GCL, *p* > 0.05) (Figure 4D). 

The number of PI positive cells showed a consistency of the damage compared to the results after 24 h (*p* > 0.05) (Figure 4C). The damage caused by Equicel^®^ or Tabotamp^®^ affected all sectional images of the z-stacks. 

### 2.5. Equicel^®^ Increased the Number of Isolectin B_4_ Positive Microglia Cells after 24 h 

To study ORC related reactions of microglia cells, the number of fluorescein isothiocyanate (FICT)-conjugated isolectin B_4_ (IB_4_) positive cells in the DG was quantified. In untreated controls, microglia cells were occasionally detected with typical ramified morphology after 24 h and 48 h (CTL 24 h: 12.5 IB_4_ positive cells/GCL, CTL 48 h: 11.0 IB_4_ positive cells/GCL) (Figure 4B,E,F). Treatment with gauze slightly increased the number of microglia over time (gauze 24 h: 14.8 IB_4_ positive cells/GCL, gauze 48 h: 25.0 IB_4_ positive cells/GCL) (Figure 4E,F). In contrast to 24 h, significantly more microglia cells were present in the gauze group than in controls after 48 h (*p* < 0.001). Equicel^®^ induced a significant increase in the number of microglia cells compared to gauze after 24 h (Equicel^®^ 24 h: 23.3 IB_4_ positive cells/GCL, *p* < 0.05) (Figure 4E). No significant difference between Equicel^®^ and gauze was found after 48 h (Equicel^®^ 48 h: 26.8 IB_4_ positive cells/GCL, *p* > 0.05) (Figure 4F). Comparable to PI results, there was no significant difference for Equitamp^®^ treatment after 24 or 48 h. In contrast to all other results, a significant reduced number of microglia cells was measured in the Tabotamp^®^ group (Tabotamp^®^ 24 h: 1.4 IB_4_ positive cells/GCL, *p* < 0.001; Tabotamp 48 h: 0.3 IB_4_ positive cells/GCL, *p* < 0.001) (Figure 4B,E,F).

### 2.6. Tabotamp^®^ Interfered with Immunofluorescence Staining

**Figure 5:**
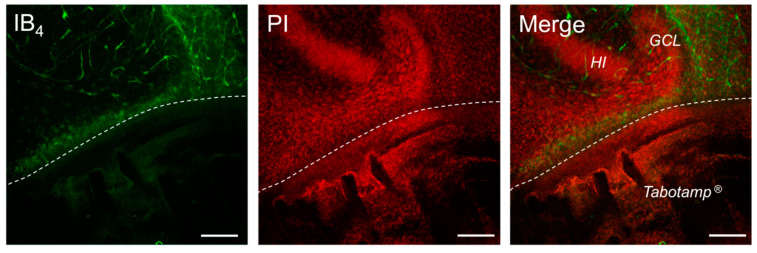
Exemplary image of an OHSC after half-side Tabotamp^®^ treatment. Damaged neuronal cells were stained with PI (red) and microglia cells as well as vascular structures with IB_4_ (green). The Tabotamp^®^ covered area was clearly distinguished (below the dotted line) from the granule cell layer (GCL) of the DG and hilus region (HI), which was still well preserved above. Notably, a clearly reduced number of IB_4_ positive cells is visible, while at the border a distinct increase of microglia cells could be observed. Bar = 100 µm.

Limited to the areas covered with Tabotamp^®^, OHSC showed an almost complete depletion of IB_4_ fluorescence signals. Inside the non-covered neuronal tissue, a clear accumulation of microglia cells was observed (Figure 5). Additional immunofluorescence staining against Iba1, an established marker for microglia cells, and further markers like NeuN and GFAP (Figure S2) showed no immunoreaction in all regions beneath Tabotamp^®^ covered areas. These results strongly supported a methodical bias related to Tabotamp^®^.

## 3. Discussion

ORC has been frequently used in neurosurgery since the 1960s, especially to stop oozing in the brain resection cavity. In clinical practice, the walls of the resection cavities are typically covered with ORC to prevent rebleeding. Whereas Equicel^®^ should be left in place after stopping the bleeding, removal of Equitamp^®^ and Tabotamp^®^ are recommended. Detachment of hemostats in intracerebral surgery strongly increases the risk of rebleeding. Therefore, in neurosurgery ORC often remain in place. However, little is known about the local effects of various ORC on neuronal tissue. Recently, we demonstrated on several monolayer cell culture systems that Tabotamp^®^ causes a strong and local cytotoxic effect and induces a strong pH drop [10].

Our here presented results show that ORC are strongly diverse in their properties and have different effects on the cells or the tissue in their vicinity. Whereas Equitamp^®^ and Tabotamp^®^ were almost completely dissolved after 48 h, Equicel^®^ swelled to a gelatine-like clot. This swelling might generate mechanical force, lead to compression and damage neuronal tissue. Even the formation of so-called textilomas was described after application of ORC during neurosurgical procedures [11,12,13]. Notably, these induced foreign body reactions in the brain can mimic recurrent intracranial tumors on MRI scans [14]. Histologically, these mass lesions were composed of not absorbed hemostat surrounded by inflammatory cellular infiltration [12]. Of the ORC investigated in this study, Equicel^®^ appeared to more likely induce a foreign body reaction due to its slower dissolution, collagen content and microglia activation in OHSC. Some of ORC reduced the pH of solutions, depending on the chemical composition and oxidation grade [15]. The bactericide and hemostatic properties of Tabotamp^®^ were associated with the strong pH reduction [3,7,8,9]. Equicel^®^ and Equitamp^®^ were proven bactericidal against gram positive and negative organisms (manufacturer’s specification). Notably and despite bactericidal effects, comparable strong pH reduction was not achieved by Equitamp^®^, and Equicel^®^ did not change the pH at all. Therefore, the bactericidal mode should be questioned for Equitamp^®^ and Equicel^®^ or parameters other than pH might be involved in bactericidal effects of these ORC. 

Previously, we showed for Tabotamp^®^ a local detachment of all tested cell types paralleled by a dose dependent increase of cytotoxicity [10]. Equitamp^®^ and Equicel^®^ also caused cell detachment albeit at a lower level indicating a decrease not only in pH value, but also in mechanical properties or other surface properties affect cell death. Cell death rates of Schwann cells increased only slightly after incubation with Equicel^®^, whereas astrocytes displayed a dose dependent enhancement of cell death rates. In contrast to the aforementioned results on Equicel^®^, incubation with Equitamp^®^ decreased cell death in all analyzed cells compared to gauze. The data hints to a pH and material dependent variable cellular response and asks for a specific use of ORC under diverse clinical situations. Depending on the localization, neuronal damage caused by ORC had far-reaching clinical consequences [16,17,18,19]. As a further step of analysis, tissue cultures such as OHSC are suitable models for studying tissue-damaging processes and neuron glia interactions [20]. Therefore, the influence of Tabotamp^®^, Equitamp^®^ and Equicel^®^ application was analyzed on cell death, microglia activation and tissue structure alterations. With PI staining [21] the damage of neuronal cells was followed in the GCL after treatment with Tabotamp^®^ and Equicel^®^ 24 and 48 h post application. Former publications reported on neuronal damage by oxidized cellulose-mediated acidity [10,22]. In contrast to these findings and in our study, the pH value remained stable even with large quantities of Equicel^®^. Probably, swelling to a gelatinous mass might be an additional reason for causing neuronal damage. This property has already been described for Tabotamp^®^ [19]. Here, the cellular damage was not only confined to the superficial cell layers but also visible in deeper layers of OHSC. However, it should be considered that tissue processing e.g., fixation or slice covering etc. might lead to a reduction of the OHSC height and therefore no reliable statement about the damage depth can be made. In contrast to Tabotamp^®^ and Equicel^®^, the cellular damage was less pronounced after incubation with gauze and Equitamp^®^. These results from OHSC underline the importance of adaptation of the ORC to the specific properties of different products by clinicians. Further examinations will be needed to evaluate whether the targeted use after gross total resection of gliomas has an influence on the development of a local recurrence. As mentioned before, so-called textilomas have been described after application of ORC. They showed a tissue reaction with accompanying phagocytic involvement and could have a damaging effect on neuronal structures [12,14,23,24,25]. Therefore, analysis of microglia reaction might be a useful parameter in context of ORC mediated neuronal injury [26,27]. In comparison to the other tested materials, Equicel^®^ led to significant increase of microglia cells after 24 h. For Equicel^®^, the manufacturer declares the addition of collagen and fibrin. These additives might affect the mechanical properties, and the rapid and massive swelling of the material might be a trigger for the microglia response. In case of gauze and Equicel^®^, the number of microglia cells grew until 48 h putatively because of the direct interaction between the material and microglia cells. However, no significant difference was observed after Equitamp^®^ treatment when compared to application of gauze after either 24 or 48 h. In contrast to the substantial neuronal damage observed after incubation with Tabotamp^®^, significantly lower numbers of microglia cells were measured. Nevertheless, a clear accumulation of microglia cells was visible at the border between the Tabotamp^®^ covered area and the non-covered area. Therefore, we asked whether a depletion of microglia cells due to the reduced pH value or other unknown mechanisms might explain this unusual phenomenon. Since no signal was detected using the additionally analyzed microglial, astroglial and neuronal markers, we concluded that Tabotamp^®^-specific factors like strong local pH drop might interfere with binding epitopes, making them more difficult or even impossible to access for lectins or antibodies. Therefore, results for Tabotamp^®^ must be viewed with caution regarding the number of microglia cells. 

## 4. Materials and Methods

All animal experiments were performed in accordance with the Policy on Ethics and the Policy on the Use by the directive 2010/63/EU of the European Parliament and of the Council of the European Union. The preparations were approved by the local authorities for care and use of laboratory animals (permission numbers: K11M1 and I11M27).

### 4.1. Cellulose Samples

Non-oxidized cellulose (cotton gauze, Fink & Walter GmbH, Merchweiler, Germany) and oxidized regenerated cellulose (ORC) samples (Equicel^®^ and Equitamp^®^, Equimedical BV, Zwaneburg, Netherlands; Tabotamp^®^, Johnson & Johnson Medical, Ethicon, Neuchâtel, Switzerland) were stamped into the specified sizes using sterile punches (Stiefel, GSK Consumer Healthcare, Dungarvan, Ireland). 

### 4.2. Analysis of Cellulose Samples in Aqueous Solution 

The 22 mm diameter samples (gauze, Equicel^®^, Equitamp^®^, Tabotamp^®^) were placed on cell culture dishes (60 cm^2^, TPP, Trasadingen, Switzerland) with 10 mL Dulbecco’s Modified Eagle’s Medium (DMEM, Thermo Fisher Scientific, Waltham, MA, USA) completed with 10% fetal calf serum (FCS, Gibco, Thermo Fisher Scientific), 2 mmol/L glutamine (Biochrom AG, Merck, Darmstadt, Germany), 100 U/mL penicillin and 100 μg/mL streptomycin (Thermo Fisher Scientific). After taking pictures, the dishes were incubated for 48 h at 37 °C and imaged again. The same composition of cell culture medium was used for cultivation of the cells, for pH measurements and for cell death analyses. Then, the dishes were incubated for 48 h at 37 °C.

### 4.3. pH Measurement

The pH measurement with increasing amounts of the different ORC was carried out in culture medium containing 44 mmol/L sodium bicarbonate with the FiveGo pH meter (Mettler Toledo, Schwerzenbach, Switzerland) at room temperature. 

### 4.4. Cell Lines 

The immortalized cells C8D1A (CRL-254, Astrocytes), RN33B (CRL-2825, Neurons) and SW10 (CRL-2766, Schwann cells) were purchased from the American Type Culture Collection (Manassas, VA, USA). SW10 and C8D1A cells were cultured in supplemented DMEM at 75 cm^2^ cell culture flasks (Sarstedt, Nümbrecht, Germany) under humidified atmosphere with 5% CO_2_ at 37 °C. RN33B cells were cultured in DMEM/F12 (1:1, Thermo Fisher Scientific) with identical supplements and conditions. The passage number of all analyzed cells was below 20.

### 4.5. Primary Cultures

Primary astrocytic cultures were prepared from p0-2 Wistar rat brains as described before [28]. Wistar neonates were purchased from the central animal facility of the medical faculty of the Martin Luther University Halle-Wittenberg.

### 4.6. Crystal Violet Staining

SW10, RN33B, C8D1A cells or primary astrocytes were incubated in culture medium for 24 h with non-oxidized cotton gauze or ORC samples. Subsequently, cell culture dishes were washed with phosphate-buffered saline (PBS, Thermo Fisher Scientific), fixed with methanol and stained with 0.5% (*w*/*v*) Crystal violet (Merck), as described recently [10]. Dishes were scanned at 600 dpi and the covered area analyzed to determine the percentage of detached cells using ImageJ/Fiji software (NIH, Bethesda, MD, USA). The ORC-covered area was set to 100%. 

### 4.7. Cytotoxicity Measurement

The 1 × 10^4^ SW10, RN33B, C8D1A cells or primary astrocytes were seeded in triplicates in black 96 well cell culture plates (Greiner Bio-One, Frickenhausen, Germany). After 24 h, different areas of cellulose samples (gauze, Equicel^®^ or Equitamp^®^; 0, 33, 55 or 100% coverage of cell layer) and the fluorescence marked DNA-binding dye CellTox-Green (1:1000, Promega, Mannheim, Germany), diluted in 200 µL culture medium were added. The fluorescence signals were measured at 485_Ex_/535_Em_ with Tecan Reader (F200PRO, Tecan, Männedorf, Switzerland) after 24 h, and the relative cell death was calculated as described previously [10]. The fluorescence signals of the completely lysed untreated cells (lysis buffer, Promega) were set to 100%. 

### 4.8. Organotypic Hippocampal Slice Cultures (OHSC)

**Figure 6:**
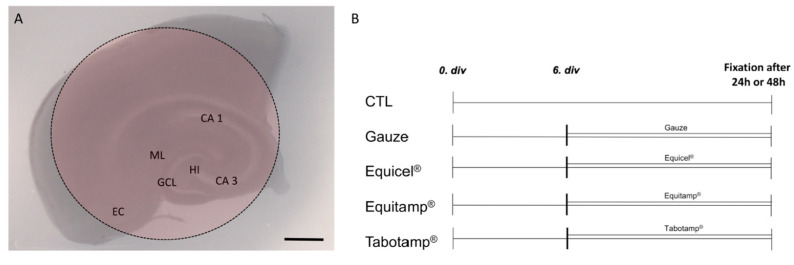
Schematic representation of an OHSC with ORC and treatment protocol. Schematic visualization of OHSC in culture. Intact cytoarchitecture of the OHSC is demonstrated with the entorhinal cortex (EC), the hippocampus with cornu ammonis (CA) subfields CA 1 and CA 3, the hilus re-gion (HI) and the dentate gyrus with the molecular layer (ML) and the granule cell layer (GCL). The red area indicates the location of the ORC (**A**). Treatment protocol of the OHSC (**B**). Bar = 1 mm.

OHSC were prepared under aseptic conditions from p8-9 Wistar rats as reported earlier [20,29] and kept in a fully humidified atmosphere with 5% (*v*/*v*) CO_2_ at 35 °C. Culture medium was replaced every second day. After six days of incubation, the slices were divided into five different treatment groups (Figure 6). OHSC were covered by 4 mm diameter pieces of Equicel^®^, Equitamp^®^, Tabotamp^®^ and gauze as a control or left uncovered as negative control (Figure 1A,B, CTL). Fixation was performed after 24 or 48 h. For all groups the culture medium was changed before starting the experiments and kept until fixation.

### 4.9. Confocal Laser Scanning Microscopy (CLSM) and Analysis of PI, NeuN, GFAP and IB_4_ Positive Cells 

Degenerated neurons were stained with 5 µg/mL of PI (Merck) 2 h before fixation. Afterwards, the OHSC were washed in PBS/Triton and incubated in normal goat serum (Sigma-Aldrich, Merck) diluted 1:20 in phosphate buffer (PB, 0.2 mol/L). Microglia cells were stained with IB_4_ (Vector Laboratories, Burlingame, CA, USA) diluted 1:50 in PBS/Triton. To validate the IB_4_ results some slices were labelled with ionized calcium binding adaptor molecule 1 antibody (Iba1, Polyclonal rabbit IgG; Thermo Fisher Scientific) diluted 1:200 in PBS/Triton and 0.5% bovine serum albumin (BSA, Sigma-Aldrich, Merck, St. Louis, MO, USA) followed by incubation with an Alexa 488 goat anti-rabbit antibody (Thermo Fisher Scientific). To investigate the effects of Tabotamp^®^ on immunofluorescence, neuronal cells were labelled with the neuronal marker (NeuN, Merck) and astrocytic cells with glial fibrillary acid protein (GFAP, BD Biosciences, CA, USA) and visualized by an Alexa 633 goat anti-mouse antibody. After staining, the slices were washed with PBS/Triton and cover slipped with fluorescent mounting medium (DAKO, Hamburg, Germany). The imaging of the OHSC was performed using a confocal laser scanning microscope (DMi8; Leica Microsystems, Wetzlar, Germany) with an excitation wavelength of 488 nm for IB_4_ and Iba1 and an emission bandpass filter of 505 to 530 nm for IB_4_ and 505 to 560 nm for Iba1, an excitation wavelength of 543 nm and an emission bandpass filter of 585 to 615 nm for PI and an excitation wavelength of 633 nm and an emission long pass filter of 650 nm for GFAP and NeuN. The images were taken using a z-scan with a section distance of 5 µm. Using Diamidine-2-phenylindol dihydrochloride (DAPI, Sigma-Aldrich) staining, the total mesh depth was displayed and thus the limits for the z-stack were determined. On average, 10–11 optical sections were obtained from one OHSC. For PI and IB_4_ analyses, only the three middle sections of an OHSC were evaluated at 200-fold magnification with a resolution of 1024 × 1024 pixels. The number of PI and IB_4_ positive cells in the DG was analyzed with MATLAB (MathWorks, Natick, MA, USA).

### 4.10. Bright-Field Microscopy

Cellular damage and cytoarchitecture were qualitatively validated by hematoxylin labelling (Roth, Karlsruhe, Germany). OHSC were washed with PBS/Triton and stained with hematoxylin, dehydrated and placed on a coverslip with Entellan (Merck). Specimens were analyzed with NanoZoomer-SQ (Hamamatsu, Iwata City, Japan). 

### 4.11. Statistical Analysis

Statistical analysis was performed with Prism 6 (GraphPad Software, San Diego, CA, USA) or SPSS software (version 25.0, IBM, Ehningen, Germany). The one-way ANOVA was used followed by Bonferroni’s test for multiple comparisons between the groups. The data are presented as mean and SEM. *p* < 0.05 was considered statistically significant. All analyses were carried out after at least three independent experiments.

## 5. Conclusions

The frequent clinical application of ORC as a heterogeneous group should consider their dramatically different impact on local pH values, dissolution time, as well as their cytotoxic properties. Equicel^®^ and Tabotamp^®^ led to a pronounced cellular damage of OHSC, whereas Equitamp^®^ causes the lowest cellular damage and cell death rate. The fast-rising number of microglia cells after incubation with Equicel^®^ indicates an early immune response. Overall, our data support a specific use of ORC in distinct clinical settings depending on its different properties. For better understanding of clinical implications of ORC further clinical studies are needed to verify and compare their complications especially in eloquent areas.

## Data Availability

Data is contained within the article and Supplementary Material (Tables S1 and S2).

## Supplementary Materials

The following are available online at https://www.mdpi.com/article/10.3390/ijms222111467/s1.
