# Supplementary material for: Variants of Oxidized Regenerated Cellulose and Their Distinct Effects on Neuronal Tissue
Source: Int J Mol Sci. 2021 Oct 25;22(21):11467. doi: 10.3390/ijms222111467 (PMC8584153; doi:10.3390/ijms222111467)
Supplement: Supplementary file 1 [file ijms-22-11467-s001.zip › Supplementary Fig 1.pdf]

Supplementary Fig. S1

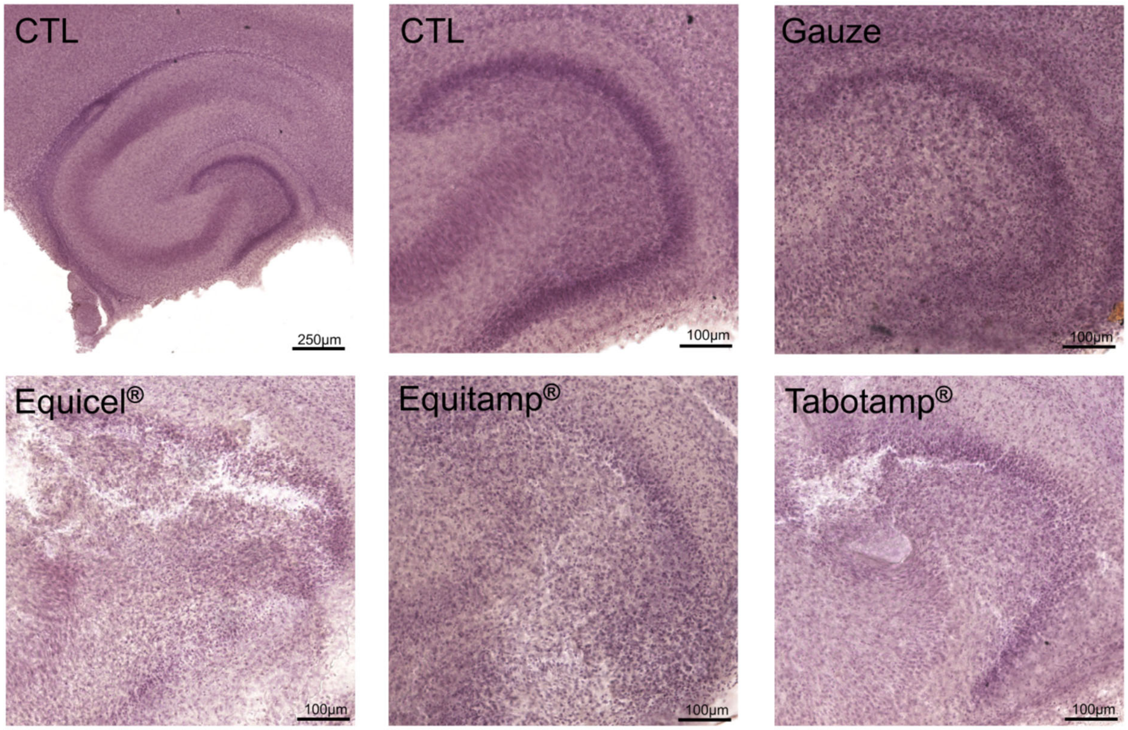

Figure S1. Hematoxylin staining of OHSC. In the control group (CTL) and after treatment with the gauze, a very well preserved cytoarchitecture is shown in which the hilus region as well as the dental gyrus are well recognizable. Especially after Equicel® and Tabotamp® treatment, numerous pycnic nuclei and a clearly changed cytoarchitecture were observed. In contrast, Equitamp® treated groups achieved a better preserved cytoarchitecture. Bars = 250 µm (left upper picture) or 100 µm.
