# Supplementary material for: Variants of Oxidized Regenerated Cellulose and Their Distinct Effects on Neuronal Tissue
Source: Int J Mol Sci. 2021 Oct 25;22(21):11467. doi: 10.3390/ijms222111467 (PMC8584153; doi:10.3390/ijms222111467)
Supplement: Supplementary file 1 [file ijms-22-11467-s001.zip › Supplementary Fig 2.pdf]

Supplementary Fig. S2

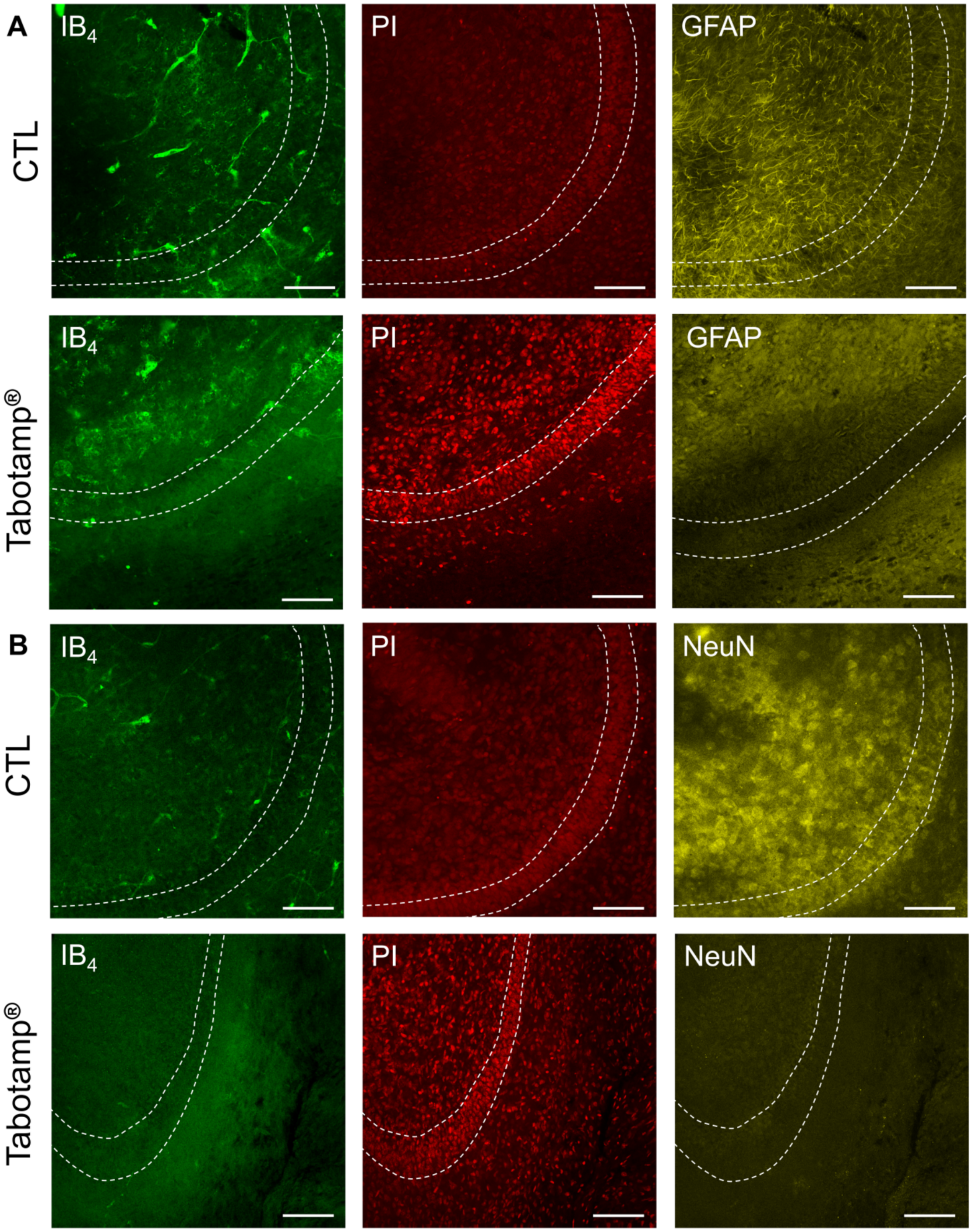

Figure S2. Triple labeling and confocal laser scanning microscopy of control and with Tabotamp® treated OHSC. Microglia are stained by IB_4 (green), damaged neurons by PI (red), astrocytes by GFAP (yellow) (**A**) and neurons by NeuN staining (yellow) (**B**). In the control groups (CTL) several microglial cells and a few PI positive cells are visible (**A, B**). Tabotamp® showed only few IB_4 positive microglia compared to CTL, as well as a clear increase in PI positive cells in the dental gyrus (DG) (**A, B**). In addition, in the CTL the typical morphology of GFAP immunoreactive astrocytes (**A**) and NeuN positive neuronal cell bodies **(B)** are shown. In the Tabotamp® treated groups, a similar picture as in the IB_4 staining was observed for the immunofluorescence of GFAP (**A**) as well as for the NeuN staining (**B**). Dotted lines mark the borders of GCL. Bars = 100 µm.
