# Supplementary material for: Variants of Oxidized Regenerated Cellulose and Their Distinct Effects on Neuronal Tissue
Source: Int J Mol Sci. 2021 Oct 25;22(21):11467. doi: 10.3390/ijms222111467 (PMC8584153; doi:10.3390/ijms222111467)
Supplement: Supplementary file 1 [file ijms-22-11467-s001.zip › Table S1.pdf]

Table S1. Impact of ORC products and gauze on cell death of Schwann cells, neuronal cells and astrocytes.

	SW10	RN33B	C8D1A	Primary astrocytes
**Treatment**	**Mean Diff.** **(95% CI) †**	**Mean Diff.** **(95% CI) †**	**Mean Diff.** **(95% CI) †**	**Mean Diff** **(95% CI) †**
**CTL vs. Gauze 33%**	-5.22 (-16.38 to 5.95)	-16.45 (-39.30 to 6.39)	-9.48 (-30.90 to 11.94)	-16.98 (-47.88 to 13.93)
**CTL vs. Gauze 55%**	-4.82 (-15.98 to 6.34)	-18.38 (-41.23 to 4.46)	**-21.82 (-43.24 to -0.40) †**	**-31.90 (-62.80 to -0.99) †**
**CTL vs. Gauze 100%**	-5.05 (-16.21 to 6.11)	-21.31 (-44.15 to 1.53)	-14.37 (-35.79 to 7.05)	**-39.15 (-70.05 to -8.25) †**
**CTL vs. Equicel 33%**	-6.72 (-17.88 to 4.45)	**-28.27 (-56.25 to -0.29) †**	**-39.27 (-60.69 to -17.85) †**	-7.58 (-38.48 to 23.32)
**CTL vs. Equicel 55%**	**-13.31 (-24.48 to -2.15) †**	**-43.33 (-66.17 to -20.48) †**	**-60.69 (-82.11 to -39.27) †**	**-41.41 (-72.31 to -10.50) †**
**CTL vs. Equicel 100%**	**-15.28 (-26.44 to -4.11) †**	**-52.15 (-75.00 to -29.31) †**	**-69.59 (-91.00 to -48.17) †**	**-51.46 (-82.37 to -20.56) †**
**CTL vs. Equitamp 33%**	3.23 (-7.86 to 14.46)	-9.96 (-32.81 to 12.88)	-1.87 (-23.29 to 19.55)	-2.16 (-33.07 to 28.74)
**CTL vs. Equitamp 55%**	1.56 (-9.60 to 12.73)	-14.90 (-37.74 to 7.95)	-2.41 (-23.83 to 19.01)	-5.57 (-36.48 to 25.33)
**CTL vs. Equitamp 100%**	-2.93 (-14.09 to 8.24)	-13.54 (-36.38 to 9.31)	-0.22 (-21.64 to 21.20)	-7.86 (-38.77 to 23.04)
**Gauze 33% vs. Gauze 55%**	0.40 (-10.77 to 11.56)	-1.93 (-24.78 to20.91)	-12.34 (-33.76 to 9.08)	-14.92 (-45.82 to 15.98)
**Gauze 33% vs. Gauze 100%**	0.18 (-11.00 to 11.33)	-4.86 (-27.70 to 17.98)	-4.90 (-26.32 to 16.52)	-22.17 (-53.07 to 8.73)
**Gauze 33% vs. Equicel 33%**	-1.50 (-12.66 to 9.66)	-11.82 (-39.79 to 16.16)	**-29.79 (-51.21 to -8.37) †**	9.40 (-21.50 to 40.30)
**Gauze 33% vs. Equicel 55%**	-8.10 (-19.26 to 3.07)	**-26.88 (-49.72 to -4.03) †**	**-51.22 (-72.64 to -29.80) †**	-24.43 (-55.33 to 6.48)
**Gauze 33% vs. Equicel 100%**	-10.06 (-21.22 to 1.10)	**-35.70 (-58.54 to -12.85) †**	**-60.11 (-81.53 to -38.69) †**	**-34.49 (-65.39 to -3.58) †**
**Gauze 33% vs. Equitamp 33%**	8.52 (-2.65 to 19.68)	6.49 (-16.35 to 29.33)	7.60 (-13.82 to 29.02)	14.81 (-16.09 to 45.72)
**Gauze 33% vs. Equitamp 55%**	6.78 (-4.38 to 17.94)	1.56 (-21.29 to 24.40)	7.07 (-14.35 to 28.49)	11.40 (-19.50 to 42.31)
**Gauze 33% vs. Equitamp 100%**	2.29 (-8.87 to 13.46)	2.92 (-19.93 to 25.76)	9.25 (-12.17 to 30.67)	9.12 (-21.79 to 40.02)
**Gauze 55% vs. Gauze 100%**	-0.23 (-11.39 to 10.93)	-2.93 (-25.77 to 19.91)	7.45 (-13.97 to 28.86)	-7.25 (-38.15 to 23.65)
**Gauze 55% vs. Equicel 33%**	-1.90 (-13.06 to 9.27)	-9.88 (-37.86 to 18.09)	-17.45 (-38.87 to 3.97)	24.32 (-6.58 to 55.22)
**Gauze 55% vs. Equicel 55%**	-8.49 (-19.66 to 2.67)	**-24.94 (-47.79 to -2.10) †**	**-38.87 (-60.29 to -17.45) †**	-9.51 (-40.41 to 21.39)
**Gauze 55% vs. Equicel 100%**	-10.46 (-21.62 to 0.71)	**-33.77 (-56.61 to -10.92) †**	**-47.76 (-69.18 to -26.35) †**	-19.57 (-50.47 to 11.34)
**Gauze 55% vs. Equitamp 33%**	8.12 (-3.04 to 19.28)	8.42 (-14.42 to 31.27)	19.95 (-1.47 to 41.37)	29.73 (-1.17 to 60.63)
**Gauze 55% vs. Equitamp 55%**	6.38 (-4.78 to17.55)	3.49 (-19.36 to 26.33)	19.41 (-2.01 to 40.83)	26.32 (-4.58 to 57.22)
**Gauze 55% vs. Equitamp 100%**	1.90 (-9.27 to13.06)	4.85 (-18.00 to 27.69)	**21.60 (0.18 to 43.02) †**	24.04 (-6.87 to 54.94)
**Gauze 100% vs. Equicel 33%**	-1.67 (-12.83 to 9.50)	-6.95 (-34.93 to 21.02)	**-24.89 (-46.31 to -3.47) †**	**31.57 (0.67 to 62.47) †**
**Gauze 100% vs. Equicel 55%**	-8.26 (-19.43 to 2.90)	-22.01 (-44.86 to 0.83)	**-46.32 (-67.74 to -24.90) †**	-2.26 (-33.16 to 28.65)
**Gauze 100% vs. Equicel 100%**	-10.23 (-21.39 to 0.94)	**-30.84 (-53.68 to -7.99) †**	**-55.21 (-76.63 to -33.79) †**	-12.32 (-43.22 to 18.59)
**Gauze 100% vs. Equitamp 33%**	8.35 (-2.81 to 19.51)	11.35 (-11.49 to 34.20)	12.50 (-8.92 to 33.92)	**36.98 (6.08 to 67.89) †**
**Gauze 100% vs. Equitamp 55%**	6.61 (-4.55 to 17.78)	6.42 (-16.43 to 29.26)	11.97 (-9.45 to 33.38)	**33.57 (2.67 to 64.48) †**
**Gauze 100% vs. Equitamp 100%**	2.13 (-9.04 to 13.29)	7.78 (-15.07 to 30.62)	14.15 (-7.27 to 35.57)	**31.29 (0.38 to 62.19) †**
**Equicel 33% vs. Equicel 55%**	-6.60 (-17.76 to 4.57)	-15.06 (-43.04 to 12.92)	**-21.42 (-42.84 to -0.01) †**	**-33.83 (-64.73 to -2.92) †**
**Equicel 33% vs. Equicel 100%**	-8.56 (-19.72 to 2.60)	-23.88 (-51.86 to 4.09)	**-30.32 (-51.74 to -8.90) †**	**-43.89 (-74.79 to -12.98) †**
**Equicel 33% vs. Equitamp 33%**	10.02(-1.15 to 21.18)	18.30 (-9.67 to 46.28)	**37.40 (15.98 to 58.81) †**	5.41 (-25.49 to 36.32)
**Equicel 33% vs. Equitamp 55%**	8.28 (-2.88 to 19.44)	13.37 (-14.61 to 41.35)	**36.86 (15.44 to 58.28) †**	2.00 (-28.90 to 32.91)
**Equicel 33% vs. Equitamp 100%**	3.79 (-7.37 to 14.96)	14.73 (-13.25 to 42.71)	**39.04 (17.63 to 60.46) †**	-0.28 (-31.19 to 30.62)
**Equicel 55% vs. Equicel 100%**	-1.97 (-13.13 to 9.20)	-8.82 (-31.67 to 14.02)	-8.89 (-30.31 to 12.53)	-10.06 (-40.96 to 20.84)
**Equicel 55% vs. Equitamp 33%**	**16.61 (5.45 to 27.78) †**	**33.37 (10.52 to 56.21) †**	**58.82 (37.40 to 80.24) †**	**39.24 (8.34 to 70.14) †**
**Equicel 55% vs. Equitamp 55%**	**14.88 (3.71 to 26.04) †**	**28.43 (5.59 to 51.28) †**	**58.28 (36.86 to 79.70) †**	**35.83 (4.93 to 66.73) †**
**Equicel 55% vs. Equitamp 100%**	10.39 (-0.78 to 21.55)	**29.79 (6.95 to 52.63) †**	**60.47 (39.05 to 81.89) †**	**33.54 (2.64 to 64.45) †**
**Equicel 100% vs. Equitamp 33%**	**18.58 (7.41 to 29.74) †**	**42.19 (19.34 to 65.03) †**	**67.71 (46.29 to 89.13) †**	**49.30 (18.40 to 80.20) †**

**Equicel 100% vs. Equitamp 55%**	**16.84 (5.68 to 28.00) †**	**37.25 (14.41 to 60.10) †**	**67.18 (45.76 to 88.60) †**	**45.89 (14.99 to 76.79) †**
**Equicel 100% vs. Equitamp 100%**	**12.35 (1.19 to 23.52) †**	**38.61 (15.77 to 61.46) †**	**69.36 (47.94 to 90.78) †**	**43.60 (12.70 to 74.50) †**
**Equitamp 33% vs. Equitamp 55%**	-1.74 (-12.90 to 9.43)	-4.93 (-27.78 to 17.91)	-0.54 (-21.96 to 20.88)	-3.41 (-34.31 to 27.49)
**Equitamp 33% vs. Equitamp 100%**	-6.23 (-17.39 to 4.94)	-3.57 (-26.42 to 19.27)	1.65 (-19.77 to 23.07)	-5.70 (-36.60 to 25.21)
**Equitamp 55% vs. Equitamp 100%**	-4.49 (-15.65 to 6.67)	1.36 (-21.48 to 24.20)	2.19 (-19.23 to 23.60)	-2.29 (-33.19 to 28.61)

Values for Tabotamp® are already published[10] and therefore not listed here. Fluorescence signal of total cell lysis was set to 100 %. The respective mean values as well as the mean difference and 95% confidence interval are shown. SW10 (Schwann cells), RN33B (neuronal cells), C8D1A (astrocytic cells). † indicates $p<0.05$ analyzed with one-way ANOVA followed Bonferroni's test.
