# Supplementary material for: Variants of Oxidized Regenerated Cellulose and Their Distinct Effects on Neuronal Tissue
Source: Int J Mol Sci. 2021 Oct 25;22(21):11467. doi: 10.3390/ijms222111467 (PMC8584153; doi:10.3390/ijms222111467)
Supplement: Supplementary file 1 [file ijms-22-11467-s001.zip › Table S2.pdf]

Table S2. Effect of different ORC and gauze on the number of PI positive neurons and IB_4 positive microglia in OHSC at 24 and 48 hours.

Treatment (PI)	Mean 1	Mean 2	Mean Diff. (95% CI) †	Treatment (PI)	Mean 1	Mean 2	Mean Diff. (95% CI) †
24 h				***48 h***			
CTL vs. Gauze	5.4	42.8	-37.4 (-156.0 to 81.1)	CTL vs. Gauze	3.4	57.6	-54.1 (-124.1 to 15.9)
CTL vs. Equicel®	5.4	256.2	**-250.8 (-349.4 to -152.2) †**	CTL vs. Equicel®	3.4	299.4	**-296.0 (-372.6 to -219.3) †**
CTL vs. Equitamp®	5.4	108.3	-103.0 (-215.2 to 9.3)	CTL vs. Equitamp®	3.4	57.3	-53.9 (-119.9 to 12.1)
CTL vs. Tabotamp®	5.4	230.4	**-225.0 (-316.0 to -134.0) †**	CTL vs. Tabotamp®	3.4	170.4	**-167.0 (-237.0 to -97.0) †**
Gauze vs. Equicel®	42.8	256.2	**-213.4 (-327.3 to -99.5) †**	Gauze vs. Equicel®	57.6	299.4	**-241.8 (-318.5 to -165.2) †**
Gauze vs. Equitamp®	42.8	108.3	-65.5 (-191.4 to 60.4)	Gauze vs. Equitamp®	57.6	57.3	0.2 (-65.6 to 66.2)
Gauze vs. Tabotamp®	42.8	230.4	**-187.6 (-295.0 to -80.2) †**	Gauze vs. Tabotamp®	57.6	170.4	**-112.9 (-182.9 to -42.9) †**
Equicel® vs. Equitamp®	256.2	108.3	**147.9 (40.5 to 255.2) †**	Equicel® vs. Equitamp®	299.4	57.3	**242.1 (169.0 to 315.1) †**
Equicel® vs. Tabotamp®	256.2	230.4	25.8 (-59.1 to 110.7)	Equicel® vs. Tabotamp®	299.4	170.4	**129.0 (52.3 to 205.6) †**
Equitamp® vs. Tabotamp®	108.3	230.4	**-122.1 (-222.5 to -21.6) †**	Equitamp® vs. Tabotamp®	57.3	170.4	**-113.1 (-179.1 to -47.1) †**
**Treatment (IB_4)**	**Mean 1**	**Mean 2**	**Mean Diff. (95% CI) †**	**Treatment (IB_4)**	**Mean 1**	**Mean 2**	**Mean Diff. (95% CI) †**
24 h				***48 h***			
CTL vs. Gauze	12.5	14.8	-2.3 (-11.4 to 6.8)	CTL vs. Gauze	11.0	25.0	**-14.0 (-23.7 to -4.3) †**
CTL vs. Equicel®	12.5	23.3	**-10.8 (-18.4 to -3.2) †**	CTL vs. Equicel®	11.0	26.8	**-15.8 (-26.5 to -5.1) †**
CTL vs. Equitamp®	12.5	16.5	-4.0 (-12.7 to 4.6)	CTL vs. Equitamp®	11.0	17.8	-6.8 (-16.0 to 2.4)
CTL vs. Tabotamp®	12.5	1.4	**11.1 (4.1 to 18.1) †**	CTL vs. Tabotamp®	11.0	0.3	**10.7 (1.0 to 20.5) †**
Gauze vs. Equicel®	14.8	23.3	-8.5 (-17.3 to 0.2)	Gauze vs. Equicel®	25.0	26.8	-1.8 (-12.5 to 8.9)
Gauze vs. Equitamp®	14.8	16.5	-1.7 (-11.4 to 8.0)	Gauze vs. Equitamp®	25.0	17.8	7.2 (-2.0 to 16.4)
Gauze vs. Tabotamp®	14.8	1.4	**13.4 (5.2 to 21.7) †**	Gauze vs. Tabotamp®	25.0	0.3	**24.7 (15.0 to 34.5) †**
Equicel® vs. Equitamp®	23.3	16.5	6.8 (-1.4 to 15.1)	Equicel® vs. Equitamp®	26.8	17.8	9.0 (-1.1 to 19.2)
Equicel® vs. Tabotamp®	23.3	1.4	**21.9 (15.4 to 28.5) †**	Equicel® vs. Tabotamp®	26.8	0.3	**26.5 (15.8 to 37.2) †**
Equitamp® vs. Tabotamp®	16.5	1.4	**15.1 (7.4 to 22.8) †**	Equitamp® vs. Tabotamp®	17.8	0.3	**17.5 (8.3 to 26.7) †**

For statistical analysis the one-way ANOVA was used followed by Bonferroni's test for multiple comparisons between the groups. The respective mean value as well as the mean difference and 95% confidence interval is shown. † indicates $p<0.05$.
